# Serial transverse enteroplasty and nipple valve construction, two life saving techniques for patients with short bowel syndrome, a report of 5 cases

**DOI:** 10.1186/s12893-021-01454-2

**Published:** 2021-12-30

**Authors:** Mojtaba Shafiekhani, Nazanin Azadeh, Kiarash Ashrafzadeh, Maryam Esmaeili, Hamed Nikoupour

**Affiliations:** 1 Shiraz Transplant Research Center, Shiraz University of Medical Sciences, Shiraz, Iran; 2 Department of Clinical Pharmacy, Faculty of Pharmacy, Shiraz University of Medical Sciences, Shiraz, Iran

**Keywords:** Autologous gastrointestinal reconstruction, Case report, Enterocutaneous fistula, Intestinal failure, Serial transverse enteroplasty, Short bowel syndrome

## Abstract

**Background:**

Various abdominal pathologies end up with surgical resection of small intestine. When the small intestine remnant is too short for adequate fluid and micronutrients absorption, short bowel syndrome is diagnosed. The disabling condition needs a multidisciplinary approach to design parenteral nutrition, care for thrombotic, hepatic and infectious complications and gradually wean the patient from parenteral nutrition. Various surgical techniques have been introduced to increase absorptive mucosa and enhance the intestinal adaptation process. Serial transverse enteroplasty and nipple valve reconstruction are among the procedures, which will be discussed in the current article.

**Case presentation:**

Herein, we presented 5 cases of short bowel syndrome as a consequence of abdominal laparotomies, patients were referred to our center to receive parenteral nutrition and to be prepared for the final autologous gastrointestinal reconstruction or intestinal transplantation, if indicated.

**Conclusion:**

Patient’s age, performance status and bowel remnant length determines the appropriate technique for autologous gastrointestinal reconstruction. Serial transverse enteroplasty is designed to increase bowel’s length by creating zigzag patterns through dilated bowel loops. Presence of ileocecal valve is crucial to delay intestinal transit time and to prevent colonic bacterial transfer to ileum. Patient’s with ileocecal valve loss benefit from creating an artificial valve, namely, nipple valve.

## Background

Intestinal failure (IF) is a state of reduced effective bowel surface to meet the minimum necessary body demands of macronutrients, electrolytes and fluids, so that intravenous fluid resuscitation or parenteral nutrition (PN) is inevitable to maintain health and prevent malnutrition [1]. Diminished bowel’s function is the consequence of an anatomic or functional loss of absorptive mucosa. In adults, the most common causes of short bowel syndrome (SBS) and consequent IF are complications of abdominal surgery due to catastrophic intestinal insults caused by trauma, volvulus, mesenteric ischemia, malignancies, enterocutaneous fistulae and extensive bowel resection due to Crohn’s disease [2]. Moreover, substantial diseased gut’s absorptive mucosa may result in functional SBS, the condition is seen in inflammatory bowel disease, radiation-induced enteritis and other enteropathies [3]. IF is a debilitating condition with various complications including liver steatosis, osteopenia, renal stones, dehydration and malnutrition, lowering the patients’ quality of life and survival [4]. After an extensive loss of bowel’s mucosa, the intestine undergoes adaptive changes to increase the absorptive surface. The changes include increased villi height and crypt depth and dilatation of the remaining intestinal segments [5]. As the adaptation process goes on, gradually the degree of diarrhea and fluid loss decreases and the patient might need less frequent or no PN. The process may continue for years [6]. Oral or enteral feeding stimulates intestinal adaptation; therefore, surgeons are widely encouraged to start feeding as soon as possible after bowel resection [7]. However, adaptive changes are not always desirable, as bowel dilatation may lead to bacterial overgrowth and dysmotility [8]. Acutely ill and metabolically unstable IF patients who have survived a complicated abdominal surgery are cared for in intestinal rehabilitation units (IRU) by a multidisciplinary team (MDT) including surgeons, pharmacists, nutritionists and various other specialists [9]. The MDT designs essential PN and hydration, manages fistulae, open abdomen, PN complications and infections until the patient becomes independent of inpatient PN or is prepared for further abdominal operations including autologous intestinal reconstruction (AGIR) or intestinal transplantation [10–12]. AGIR refers to various surgical techniques to enhance intestinal absorptive mucosa in order to minimize dysmotility and the need for intestinal transplantation [13]. Although still debated, AGIR is reserved for patients who despite intensive medical efforts, are not able to attain a PN-free life [8]. Serial transvers Enteroplasty (STEP) is a relatively new AGIR technique with 15 years of experience. The technique is designed to lengthen the intestine by creating zigzag lumens in dilated bowel segments [14]. In previous studies, STEP procedure has shown promising results as an ultimate technique to manage SBS. Various studies have reported 20–60% bowel lengthening by performing STEP, as well as an approximate 43% successful rate to wean the patients from PN [15, 16].

Ileocecal valve (ICV) plays a notable role in delaying intestinal transit time and preventing colonic bacterial transfer to the small intestine. Patients with ICV loss benefit from artificial valve reconstructions. Nipple valve is created by intussuscepting small intestine to colon. The valve delays distal intestinal motility, subsequently reserves more time for absorption [17].

In this report, we briefly review the history and management of 5 patients with SBS and IF, for whom STEP or nipple valve creation was done in the Abu-Ali Sina Organ Transplnat Hospital, Shiraz, Iran. The hospital is the unique referral center for IF patients in Middle East.

## Case presentation

### Case 1

#### History and presentation

**Fig. 1:**
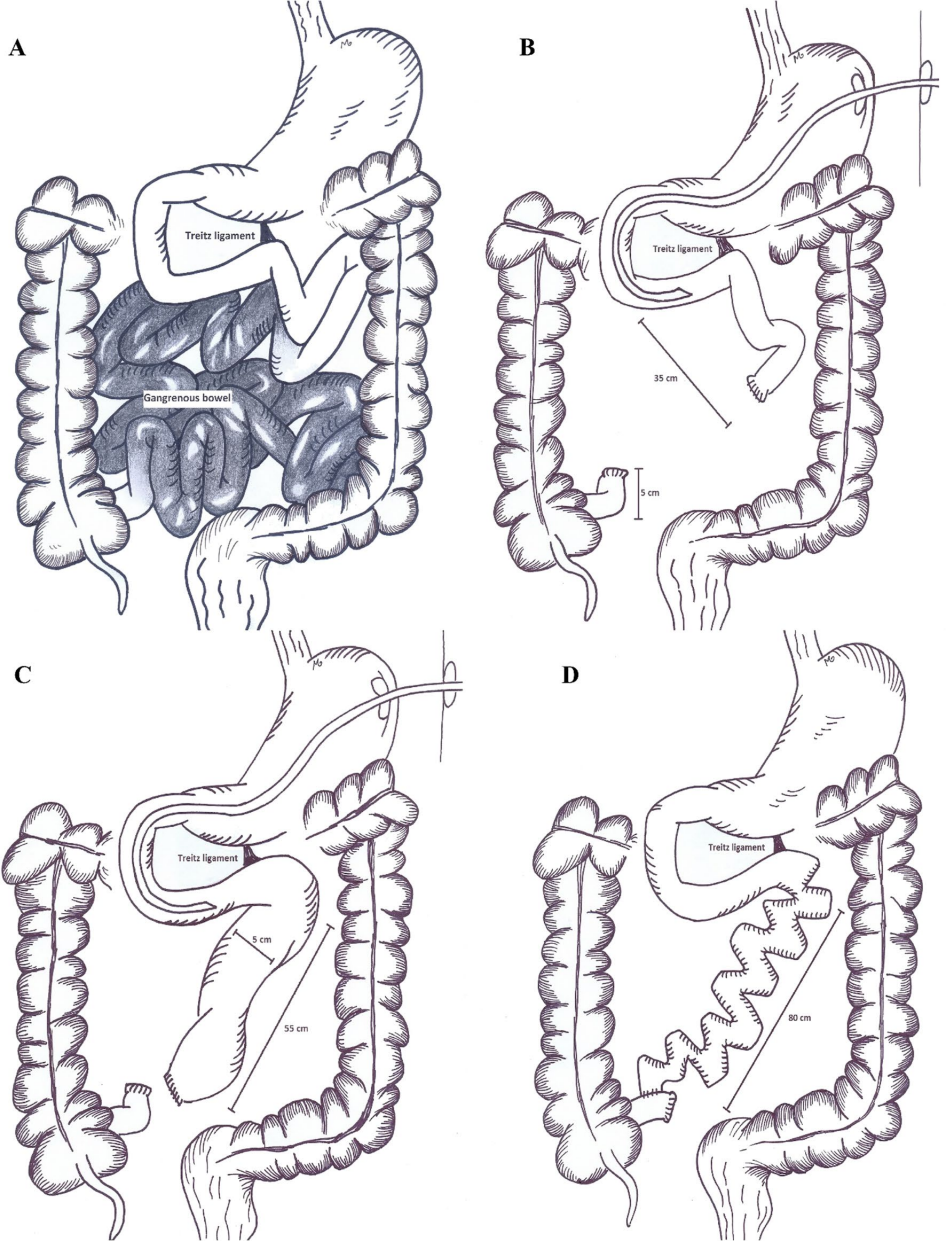
A significant length of the small bowel was gangrened (**A**) which was resected (**B**). After a while, the proximal segment was dilated (**C**). By the aid of the STEP procedure, the remaining bowel was lengthened to 80 cm (**D**)

A 58-year-old male patient was admitted with mesenteric ischemia in a local hospital. The patient had immediately undergone open laparotomy, assuming that the remaining bowel was too short in length (Fig. 1A), the surgeon had decided to close the abdomen without a certain intervention.

#### Management

The next day, our team was called and attended the patient for laparotomy. The gangrened bowel sections were resected and the viable bowel remnant measured 35 cm distal to ligament of Treitz, as well as 5 cm of remaining ileum (Fig. 1B). A gastroduodenostomy tube was inserted and the patient was transferred to IRU, where he received PN for 8 weeks. At the end of this period, a radiologic contrast study revealed dilatation of the proximal section of the bowel, for which he was scheduled to undergo an AGIR operation. During the recovery period, the length of the bowel had increased to 55 cm (Fig. 1C), which was further increased to 75 cm with STEP procedure. Considering the 5 cm ileal remnant, the final length of the remaining bowel was 80 cm at the end of the procedure (Fig. 1D).

#### Outcome

By preserving bowel’s continuity and patient’s tolerance of oral nutrition, he was discharged from the hospital 10 days later to the last surgery. On the time of preparing the article (22 months following discharge date) the patient had no complaint or no need to be admitted in the hospital due to IF symptoms and signs.

### Case 2

#### History and presentation

**Fig. 2:**
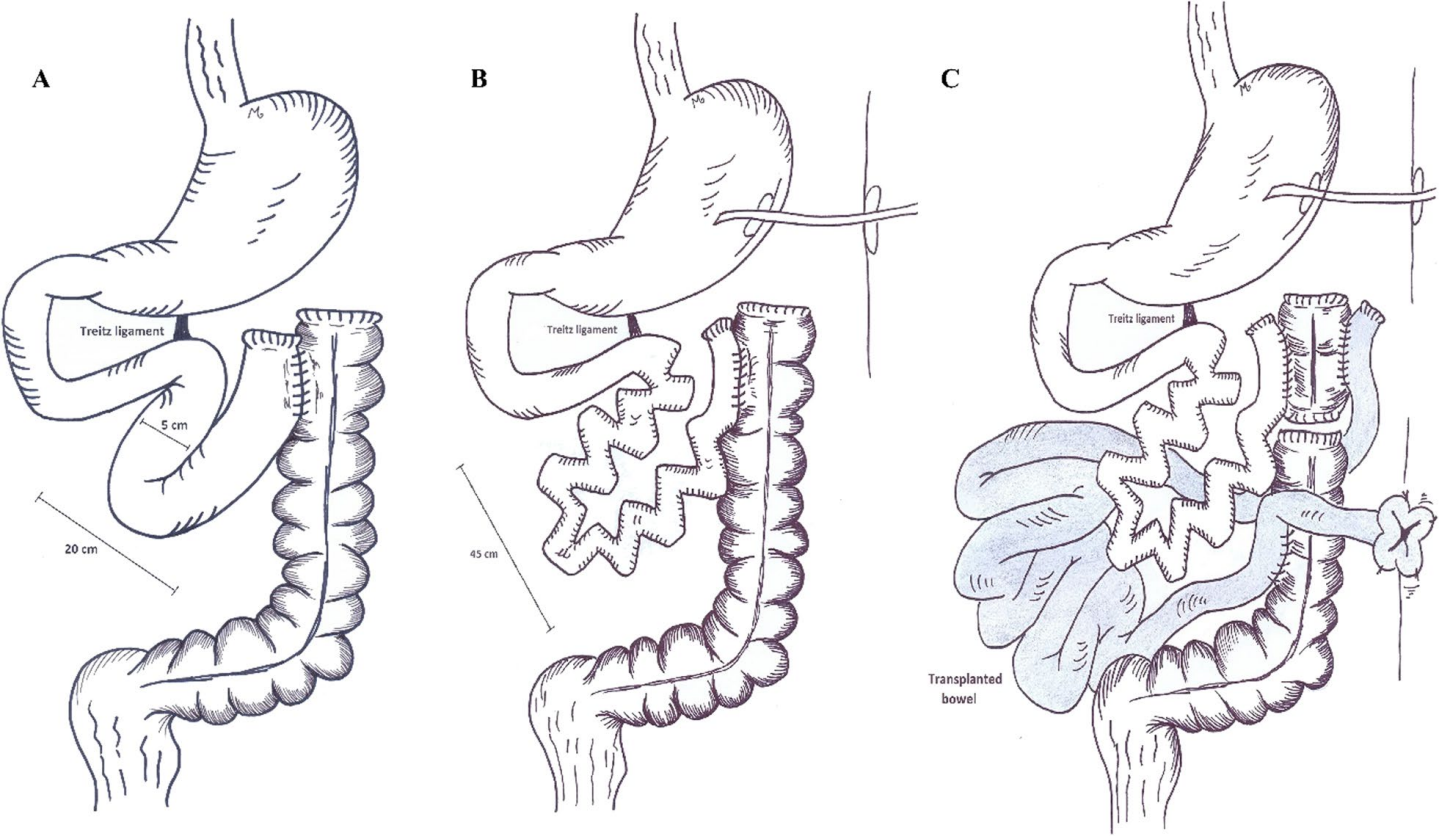
The patient was referred after extensive resection of small bowel (**A**), with the aid of the STEP procedure, the bowel’s length was increased to 45 cm (**B**), due to failure of AGIR, a small intestine transplantation was done (**C**)

In a local hospital, a 47-year-old male patient had undergone open laparotomy and resection of gangrened bowel and anastomosis of viable bowel segments due to mesenteric ischemia. At the end of the procedure, the remaining bowel measured 20 cm including left colon (Fig. 2). The patient was then referred to our center.

#### Management

A radiologic contrast study was done which revealed the bowel diameter to be 5 cm; therefore, the patient was a candidate for the STEP procedure. While exploring the abdomen in the OR, we estimated the remaining bowel’s length to be 20 cm. We performed STEP for duodenum and the 20 cm long remnant bowel. The bowel’s length was increased to 45 cm with the procedure. Afterwards, the patient received PN for 1 month and was dismissed from IRU. During the upcoming months, he was frequently admitted with dehydration. Consequently, he was put on the waiting list for small intestinal transplantation.

#### Outcome

Eventually, he underwent isolated small intestinal transplantation, which was uneventful for next 6 months. At the end of the 6th month, the patient was admitted with fungal infection of the transplanted organ, which led to sepsis and death.

### Case 3

#### History and presentation

A 36-year old lady was transferred to our center with internal bowel herniation and in an unstable hemodynamic state. Patient’s past medical history included liver transplantation due to primary sclerosing cholangitis with Roux-en-Y common hepatic duct anastomosis, 4 years prior to the current admission. She had no history of concurrent inflammatory bowel disease.

#### Management

**Fig. 3:**
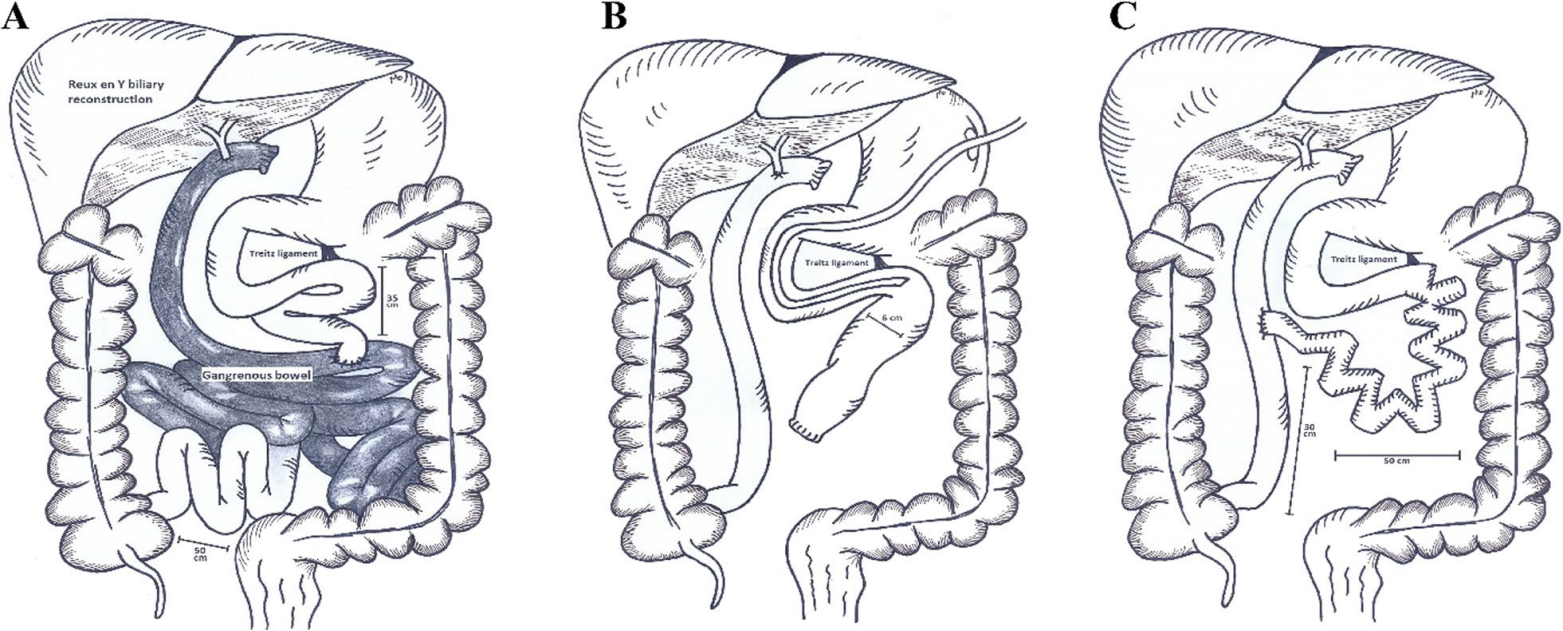
The liver recipient was admitted with internal bowel herniation and consequent bowel gangrene (**A**), the gangrened segments were immediately resected, the bile duct was anastomosed to ileum and a gastroduodenostomy tube was inserted (**B**). A week later, STEP was done on dilated remaining bowel (**C**)

She was immediately transferred to OR for an emergent laparotomy. The gangrened bowel segments were resected and the hepaticojejunostomy was taken down. With the aid of Cattell–Braasch maneuver and in a retro-colic position, the bile duct was anastomosed to the ileum. The proximal of the remaining bowel which had a diameter of 6 cm was closed and a gastroduodenostomy tube was inserted (Fig. 3). At the end of the procedure, the remnant of bowel measured 35 cm from ligament of Treitz to the jejunojejunostomy anastomosis site and 50 cm from anastomosis site to the ileocecal valve. The patient was then transferred to the intensive care unit, where the immunosuppressive medications (Tacrolimus, Prednisolone) were continued. A week later, in a stable hemodynamic state, she underwent another laparotomy to lengthen the intestinal remnant. The STEP procedure was done on the proximal segment of the bowel, increasing its length from 35 to 50 cm. Furthermore, the segment was anastomosed to the highest possible side of the Roux limb.

#### Outcome

On the 5th post-operation day, oral nutrition was started for the patient and 5 days later, she was dismissed from the hospital. In the most recent follow up, after 15 months of being discharged from hospital we assessed that the adaptation process helped the patient have a relative acceptable quality of life.

### Case 4

#### History and presentation

In a local hospital*,* a 71-year old diabetic patient had undergone resection and anastomosis of gangrened small intestine segments due to mesenteric ischemia. The patient was later referred to our center with enterocutaneous fistula.

#### Management

**Fig. 4:**
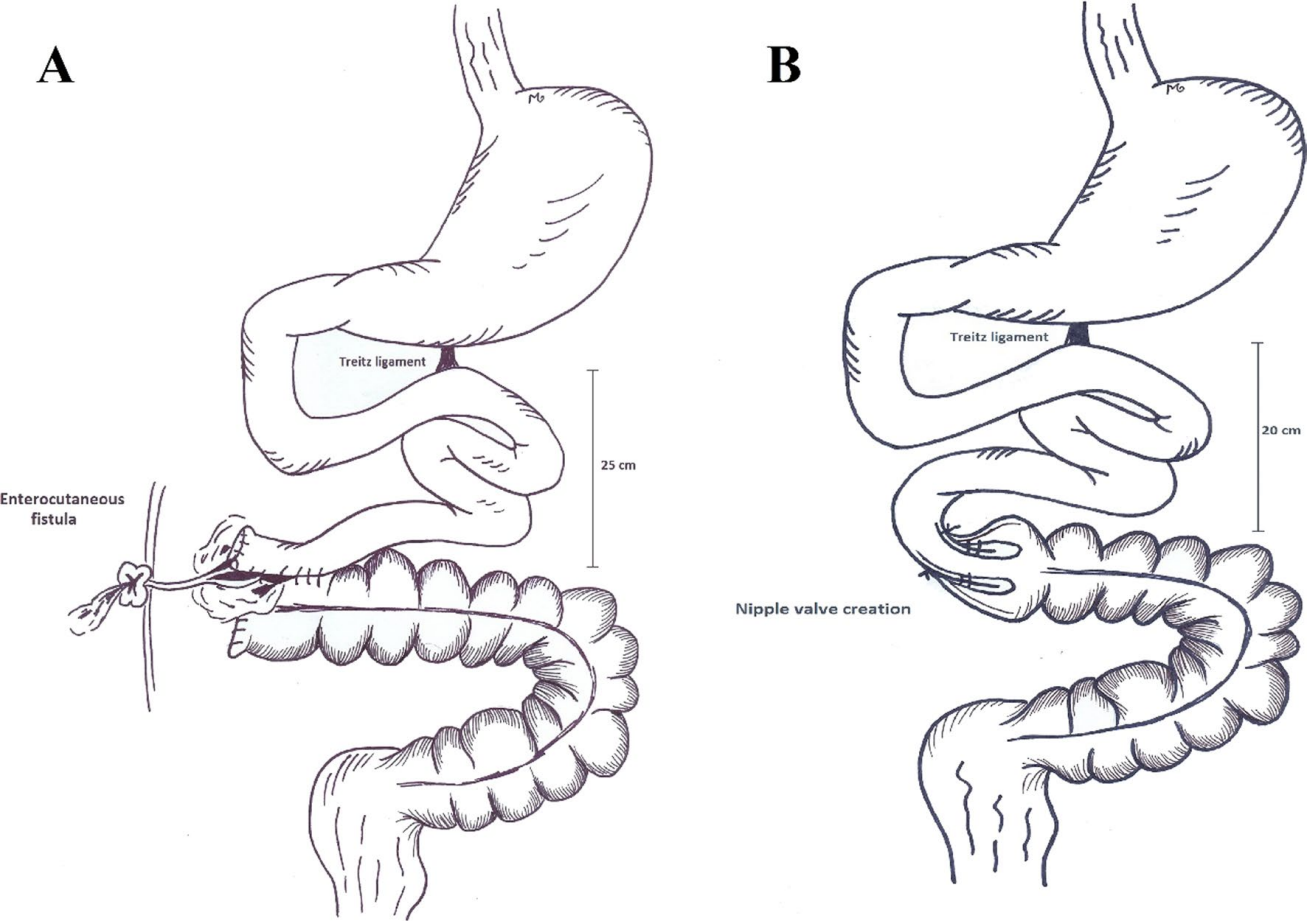
The patient was transferred to our center after resection of a considerable length of bowel and with an enterocutaneous fistula (**A**). After resection, we created a nipple valve for the patient (**B**)

During the next month, we managed the open abdomen and the patient received PN while instructed with nil per os (NPO) order. On the 35th day, the fistula had already been closed and in addition to PN, oral nutrition was started for him. Eight weeks after the primary operation, the patient was scheduled for AGIR. During the abdominal exploration, the length of the bowel remnant to the fistula was estimated to be 25 cm. After resection, 20 cm of bowel and the descending colon were the remaining segments (Fig. 4). Considering the fact that the patient was not an ideal candidate for small intestine transplantation and he would be dependent on daily PN, we decided to create a nipple valve by intussuscepting the small intestine to colon. Therefore, bowel’s continuity was preserved. Additionally, a gastrostomy tube was inserted as a vent.

#### Outcome

On the 5th post-operation day, the patient tolerated oral nutrition. During the next month, PN was gradually tapered to the lowest necessary frequency and he was discharged from the hospital with a weekly need for PN and after 2 months the patient needed no more PN. Later, patient was admitted with dehydration and severe diarrhea which was managed by fluid resuscitation and high dose loperamide for 1 month. During the past 6 months and until the latest follow up, he has gained weight and complains of no more diarrhea with 2 mg of Loperamide, administered three times in the day.

### Case 5

#### History and presentation

A-52-year old lady was admitted in a local hospital with small bowel ischemia and had undergone resection of the gangrened bowel segments with creation of an ostomy. She was then transferred to our IRU to receive PN.

#### Management

**Fig. 5:**
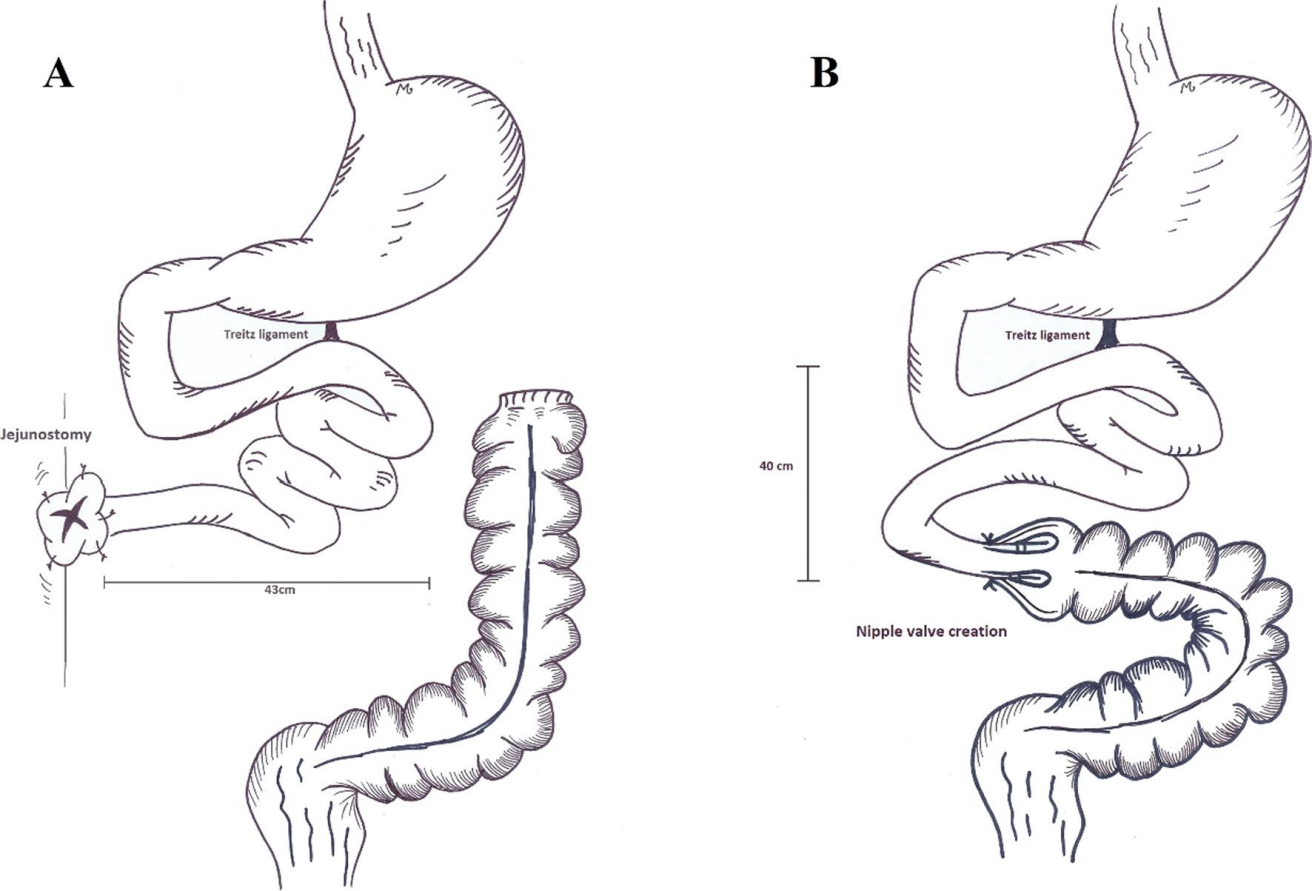
The patient was referred after extensive resection of gangrened bowel and creation of a jejunostomy (**A**). During AGIR, a nipple valve was created (**B**)

After 8 weeks of ostoma care and receiving PN as well as regular oral nutrition, she was scheduled for AGIR. A nipple valve was created in the 43 cm long remaining bowel and a gastrostomy was inserted as a vent (Fig. 5).

#### Outcome

The patient received PN for 4 weeks following surgery and was dismissed from the hospital at the end of the first post-operation month. 4 months after discharge he was once admitted with diarrhea and bacterial overgrowth which was managed by antibiotics. In the most recent follow up, after 10 months of surgery she had a satisfying quality of life without any symptoms regarding IF.

## Discussion and conclusion

SBS is a debilitating condition with considerable disturbance of patients’ quality of life and challenging management. Multidisciplinary teams are responsible to design a management plan for patients and to facilitate the small intestinal adaptation process. Once the small intestine fails, transplantation may be indicated. Considering the shortage of organs and transplantation risks, the team seeks solutions to avoid transplantation. In experienced hands, surgical techniques to lengthen the small intestinal absorptive mucosa are promising in alleviating the SBS condition [18]. To date, several small intestinal lengthening procedures have been invented and practiced worldwide. In 1980, Dr. Bianchi introduced the longitudinal intestinal lengthening and tailoring (LILT) procedure in pigs [19]. The technique was later widely practiced for SBS patients, particularly in the pediatric field. During the LILT procedure, the surgeon divides the intestine into two longitudinal halves, sutures the longitudinal sides and then reconnects the created segments to lengthen the intestine to two times as the primary length. Consequently, the final intestinal diameter is reduced to half [20]. In 2003, serial transverse enteroplasty (STEP) was introduced as a simpler method to achieve bowel lengthening by creating zigzag patterns in dilated bowel loops [14]. Since then, LILT and STEP have both been applied in IF centers with promising results. In a systematic review by Frongeia et al., both techniques were concluded to be as effective in intestinal lengthening and gaining relief of IF complications. However, STEP tends to have a slightly lower mortality rate and progression to transplantation [21]. In 2019, Shah et al., reported their 10-year experience in surgical management of SBS. The overall outcome of both techniques were similar in their follow up [22]. However debated, still the choice of the procedure mainly depends on surgeon’s experience. In our center, we are more experienced with STEP, as a less complicated procedure. Concerning of future stricture formation after LILT with our previous experiences, we prefer to perform STEP for patients with dilated bowel loops.

In this report, first we presented a case of considerable bowel shortness after resection due to mesenteric ischemia, who later underwent STEP procedure to lengthen the remaining intestine. The patient had initially underwent an exploratory laparotomy, fearing of short length of bowel remnants, the surgeon had decided to take no action. We suppose that with advances in AGIR techniques, hesitating to resect the gangrened bowel segments and delaying the procedure was an irrational choice. As, delayed resection of gangrened bowel segments is associated with higher mortality rate [23]. In presence of bowel gangrene, anastomoses will more frequently be accompanied by complications such as anastomosis leakage and fistula formation which contribute to further bowel shortening and consequent burdensome AGIR. Additionally, creating ostomies will bring about skin irritation and an additional bowel length loss after their closure [24]. Hence, we recommend closing the bowel ends and waiting for adaptive bowel dilatation. A gastroduodenostomy tube is a desirable choice for decompression of gastric secretions [25]. In this case, we observed both lengthening and dilatation of the bowel remnants during the adaptive period. When in contrast study the dilated bowel segments had reached 4 cm in diameter, the patient was scheduled for STEP procedure. The gastroduodenotomy tube was removed 1 month later.

The ileocecal valve (ICV) prolongs the intestinal transit time, allowing more time for absorption. Besides, the valve prevents bacterial backwash from colon to ileum. The absence of ICV might result in ileal bacterial overgrowth and exacerbation of existing malabsorption [26]. Therefore, preserving the ICV in cases of small bowel resection helps reduce complications of SBS [27]. Furthermore, the duration of PN dependence is associated with remaining small bowel length and presence of ICV [28]. Absence of ICV also increases the need for repeated STEP [29]. The second presented case, discusses a patient with extremely short bowel remnant after resection of gangrened segments, who despite undergoing STEP needed an intestinal transplantation due to several admissions with dehydration and electrolyte imbalance. In cases of SBS, recurrent dehydration episodes, developing PN associated liver injury and repeated central line infection and thrombosis are among the indications of small intestinal transplantation [30]. For patients with viable bowel measuring less than 40 cm and loss of ileocecal valve, we recommend considering small intestine transplantation as the ultimate treatment strategy. Even additional duodenal lengthening via STEP procedure could not save the 2nd presented patient from repeated dehydration episodes. Further bowel lengthening techniques including LILT was considered for the patient, however little chance of living a PN-free life was expected in a condition with extreme short length of bowel. Meanwhile she was put on transplantation waiting list as a treatment strategy to finalize patient’s discharge from hospital. Complications of PN, repeated admissions due to dehydration and delayed bowel transplantation adversely affected the transplantation outcome. In our country, home PN is not practiced; therefore, we recommend intestinal transplantation as the primary management strategy for patients with extremely SBS.

In the 3rd case, we presented a liver recipient lady with Roux-en-Y reconstruction who lost a considerable length of her intestines due to internal herniation and consequent gangrene. In assessment of abdominal pain in patients who have undergone Roux-en-Y reconstruction, possibility of internal herniation should always be considered and if indicated, patients should immediately undergo exploratory laparotomy to prevent bowel gangrene [31]. Approach to abdominal pain in organ recipients is not considerably different from normal population. Therefore, in cases of acute abdomen, emergent laparotomies should not be postponed until the patients are referred to organ transplantation centers [32]. In the absence of peritonitis and evidences of abdominal contamination; while bowel loops were dilated, we performed an early STEP to regain intestinal autonomy. We avoided anastomosis creation in the first surgery, considering the fact that the patient was unstable and immunosuppressed. Since the immunosuppressant agents are absorbed in duodenum and jejunum, we inserted a gastroduodenostomy tube to provide a safe route for medications. With the aid of Cattell–Braasch maneuver, a tension free biliary anastomosis was provided.

The 4th case discusses a 72-year-old patient who had underwent immediate resection of gangrened bowel segments and anastomosis of the viable tissue due to mesenteric ischemia. As earlier mentioned, in cases of bowel gangrene primary anastomosis might be complicated by anastomosis leakage and fistula, as what this patient experienced. With the lack of ileocecal valve and the viable bowel measuring 20 cm, small intestine transplantation was the optimal treatment strategy. However, considering patient’s age and performance status, he was not an appropriate candidate for transplantation. By creating a nipple valve, the patient was less frequently dependent on PN and his quality of life appreciably improved. It seemed that the nipple valve helped the patient to be discharged from hospital with an extremely short length of small bowel. Therefore, the method of AGIR differs based on patient’s performance status.

In the 5th case, we presented a lady with a stoma and closure of transverse colon after primary resection and anastomosis. Considering the fact that most patients are referred to IRUs after primary operations, the MDT should be prepared for intestinal reconstruction in various conditions, namely in presence of stomas. For patients with stomas, regular diet is started as soon as possible and an expert nurse cares for the stoma. Besides, the patient received PN for 8 weeks after the primary operation. During the intestinal reconstruction operation, 43 cm of bowel was remaining and the ileocecal valve was lost; therefore, we decided to create a nipple valve by everting 3 cm of jejunum and telescoping the its tip to the colon. As the final step, the base of the nipple was anastomosed to colon. A gastroduodenostomy tube was inserted as a vent. The patient was transferred to IRU and as soon as he could obtain 60% of his daily calorie form oral nutrition, PN was tapered. With no nipple valve creation, the patient would have been a candidate for small intestine transplantation. In our center, we have adopted the method of nipple valve for patients with 40–80 cm bowel remnant with concurrent ileocecal valve loss and no bowel dilatation.

In the current manuscript we reviewed 5 cases of SBS as a consequence of laparotomies. Adopting the STEP procedures helped 2 patients to be gradually weaned from PN; however one patient with extremely short bowel length and ICV absence did not benefit from the procedure. Nipple valve reconstruction was approached for 2 patients lacking ICV, with acceptable surgery outcome.

## Data Availability

All data generated or analysed during this study are included in this published article.

## Abbreviations

AGIR: Autologous gastrointestinal reconstruction
ICV: Ileocecal valve
IRU: Intestinal rehabilitation unit
LILT: Longitudinal intestinal lengthening and tailoring
NPO: Nil per os
OR: Operation room
PN: Parenteral nutrition
STEP: Serial transverse enteroplasty
SBS: Short bowel syndrome
